# Frugal fat or munificent muscle: genomic imprinting and metabolism

**DOI:** 10.1186/s12915-014-0104-2

**Published:** 2014-12-31

**Authors:** David Haig

**Affiliations:** Department of Organismic and Evolutionary Biology, Harvard University, 26 Oxford Street, Cambridge, MA 02138 USA

## Abstract

Variation in body composition is a popular obsession. The culturally ‘ideal’ body type is light on fat and heavy on muscle but the human population is collectively laying on fat. A new study finds antagonistic effects of two imprinted genes, *Grb10* and *Dlk1*, on body composition in mice. These findings pose the question whether there is an evolutionary conflict between genes of maternal and paternal origin over the optimal proportions of body fat and lean muscle mass.

See research article: http://www.biomedcentral.com/1741-7007/12/99

Organisms evolve to maximize their genes’ chances of leaving descendant copies. In pursuit of this goal, some species live fast and die young while others adopt a more sedate pace of life. Life-history theory attempts to understand ecological factors that shape strategic allocations between size versus number of offspring, reproductive effort versus body maintenance, early versus delayed reproduction, and making the best of current opportunities versus preparing for an uncertain future. A major strategic decision involves the relative proliferation of cells contributing to muscle mass (conferring superior earning capacity but increased maintenance costs) and fat mass (a load to be carried but insurance against hard times). Antagonistic effects of *Grb10* and *Dlk1* appear to modulate this trade-off between productive investment and precautionary savings [1]. Of particular interest, these genes are oppositely imprinted: *Grb10* is expressed from the allele a mouse inherits from its mother, but not the allele it inherits from its father, whereas *Dlk1* is expressed when inherited from fathers but not mothers.

## Jack Sprat could eat no fat, his wife could eat no lean

**Figure 1.**
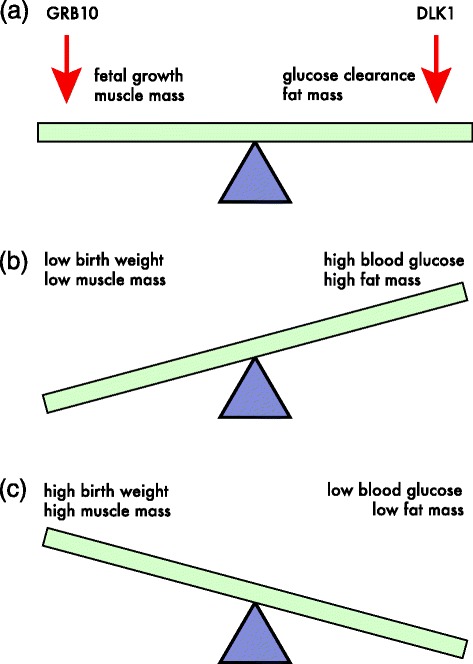
**Antagonistic effects of Grb10 and Dlk1. (a)** Maternally expressed *Grb10* and paternally expressed *Dlk1* have opposing effects on fetal growth, fat mass and muscle fat. **(b)** Inactivation of *Dlk1* results in low birth weight and muscle mass but high fat mass and blood glucose. **(c)** Inactivation of *Grb10* results in the opposite set of phenotypes.

Developmental decisions about proliferation of different cell types may have long-lasting consequences. Mice with inactivation of the expressed maternal copy of *Grb10* are born large and develop into lean adults whereas mice with inactivation of the expressed paternal copy of *Dlk1* are born small and develop into obese adults (Figure 1). Mice with inactivation of both genes resemble mice with only *Grb10* inactive. Therefore, *Grb10* and *Dlk1* appear to act in a common pathway with *Grb10* acting downstream of *Dlk1* [1]. These findings add to evidence that *Grb10* inhibits, and *Dlk1* promotes, proliferation and differentiation of muscle [2,3]. The situation with respect to adipogenesis is less clear. Although several earlier studies have concluded that *Dlk1* inhibits the recruitment of fat cells, a recent study found that adipogenesis was unaffected by overexpression of *Dlk1* [4].

Mice with fat-specific ablation of *Grb10* have more lipid per adipocyte without a change in adipocyte number [5]. Obesity in these mice seems to contradict the lean phenotype of mice who inherit a disrupted maternal copy of *Grb10* [1]. The observations would be reconciled if inherited deletions cause reduced recruitment of adipocytes but fat-specific deletions cause increased accumulation of lipid in already differentiated adipocytes. This would imply that *Grb10* inhibits recruitment in preadipocytes before the fat-specific deletion has effect, or that the leanness of mice with inherited deletions is an indirect consequence of effects in non-adipose tissue.

The kinship theory of genomic imprinting predicts that maternally expressed genes (MEGs) should benefit the individual in which they are expressed at a cost to patrilineal kin or impose individual costs for the benefit of matrilineal kin (including mothers). Conversely, paternally expressed genes (PEGs) are predicted to benefit an individual at a cost to matrilineal kin or impose individual costs for the benefit of patrilineal kin [6]. The heavy birth weight of mouse pups with an inactive maternal copy of *Grb10* and the low birth weight of pups with an inactive paternal copy of *Dlk1* are consistent with these predictions because the MEG inhibits fetal growth for maternal benefit whereas the PEG promotes fetal growth at maternal cost. In this case, imprinted genes mediate an evolutionary conflict over the trade-off between offspring size and number.

Contrasting effects of *Grb10* and *Dlk1* on muscle and fat hint at evolutionary conflict between MEGs and PEGs over body composition, with MEGs favoring more fat and PEGs favoring more muscle. Consistent with this pattern, Prader-Willi syndrome, which is caused by the failure to express one or more PEGs on human chromosome 15, is associated with morbid obesity and low muscle mass. Although *Grb10* behaves as a MEG in muscle and fat, it behaves as a neuronal PEG. This switch from maternal-specific to paternal-specific expression in the central nervous system is recapitulated in culture as mouse embryonic stem cells differentiate into neurons [7].

Evolutionary scenarios that invoke differential consequences of body composition for matrilineal and patrilineal kin can be constructed to explain why MEGs and PEGs might favor different allocations between muscle and fat or between brain and brawn. As an example, if (i) mice occupy territories with matrilineal kin and (ii) groups of smaller, plumper mice better survive periods of famine but (iii) greater muscle mass confers an advantage in competition for food within groups, then MEGs should favor more fat and less muscle than PEGs. However, we need to know much more about patterns of cooperation and competition among kin in wild mice before such scenarios can be as compelling as the simple story of maternal-paternal conflict over fetal growth.

## Alternative energy supplies

Imprinted genes influence not only the balance of tissues within bodies but also basic metabolic parameters. Choices of fuel - whether to burn or stored fat and whether to use amino acids for gluconeogenesis or to build proteins - are implicated in major life-history trade-offs, including those between investment in present reproduction and precautionary savings. One aspect of murine metabolism in which imprinted genes play a significant role is the control of thermogenesis (heat production) by brown adipose tissue (BAT).

Huddling for warmth is a simple cooperative behavior in which the costs of heat production are borne by individuals but benefits are shared. MEGs are predicted to favor greater contributions to the collective good and PEGs to favor free-riding under the assumption that mice preferentially huddle with kin, such as sibs from multiple paternity litters, who are closer relatives on the maternal side than on the paternal side [8]. Thus, *Grb10* (a MEG) promotes thermogenesis [5] whereas *Dlk1* (a PEG) inhibits thermogenesis and reduces BAT mass at weaning [9]. Overexpression of *Dlk1*, however, is associated with increased BAT in the immediate postnatal period [9]. The body mass and huddling ability of pups change dramatically in the period from birth until weaning. It is possible that the different effects of *Dlk1* on early and late BAT reflect a reversal in the ratio of individual to communal benefits of heat production.

GRB10 and DLK1 are also implicated in the regulation of blood glucose levels. Lean mice with an inactivated maternal copy of *Grb10* exhibit increased glucose uptake by skeletal muscle and rapid clearance of a glucose load [1]. By contrast, mice with fat-specific ablation of *Grb10* are obese and glucose intolerant [5]. This suggests that GRB10 normally reduces glucose uptake in skeletal muscle but increases glucose uptake in one or more other tissues. Parent-of-origin effects were detected at human *GRB10* in a meta-analysis of genome-wide association studies of insulin response to an oral-glucose tolerance test. One SNP (rs933360-A) was associated with lower fasting glucose and enhanced insulin sensitivity when maternally inherited but higher fasting glucose and reduced insulin sensitivity when paternally inherited [10].

## Evolutionary metabolism

Organisms must prudently manage their portfolio of investments over the course of a lifetime. Strategic choices at the organismal level must emerge from information-processing within cells. Each cell must not only manage its own metabolism, second by second and minute by minute, but also coordinate its activities over much longer periods with many other cells of the same and different types. Hormones and other circulating factors provide cues that allow cellular behavior to be matched to organismal goals. Growth hormone, for example, modulates fuel choice between fats and protein (via gluconeogenesis). Increased reliance on lipids to conserve protein would be favored both during rapid linear growth and during malnutrition but the adaptive tissue-specific responses in these two situations should differ markedly as elevated growth hormone is integrated with other cues.

A model of organisms as well-designed machines optimizing well-defined fitness functions is implicit in much of traditional physiology and modern systems biology (as well as in the preceding paragraph). But antagonistic effects of MEGs and PEGs suggest that genes of maternal and paternal origin have had different fitness functions with respect to body composition and other metabolic parameters. Such divergence of fitness functions occurs when an organismal phenotype mediates fitness trade-offs among kin [6]. A challenge for future evolutionary study is to understand the nature of these trade-offs.
